# Reactive T Cells in Convalescent COVID-19 Patients With Negative SARS-CoV-2 Antibody Serology

**DOI:** 10.3389/fimmu.2021.687449

**Published:** 2021-07-12

**Authors:** Sophie Steiner, Tatjana Schwarz, Victor M. Corman, Franziska Sotzny, Sandra Bauer, Christian Drosten, Hans-Dieter Volk, Carmen Scheibenbogen, Leif G. Hanitsch

**Affiliations:** ^1^ Charité – Universitätsmedizin Berlin, Corporate Member of Freie Universität Berlin and Humboldt Universität zu Berlin, Institute of Medical Immunology, Augustenburger Platz 1, Berlin, Germany; ^2^ Charité –Universitätsmedizin Berlin, Corporate Member of Freie Universität Berlin and Humboldt-Universität zu Berlin, Institute of Virology, and German Centre for Infection Research (DZIF), associated partner, Charitéplatz 1, Berlin, Germany; ^3^ Berlin Institute of Health at Charité –Universitätsmedizin Berlin, Berlin, Germany; ^4^ Berlin Institute of Health at Charité -Universitätsmedizin Berlin, BIH Center for Regenerative Therapies (BCRT), Charitéplatz 1, Berlin, Germany; ^5^ Berlin Center for Advanced Therapies (BeCAT), Charité - Universitätsmedizin Berlin, Berlin, Germany

**Keywords:** coronavirus disease 2019 (COVID-19), T cell response, seronegative, antibody response, human endemic coronavirus 229E (HCoV-229E), human endemic coronavirus OC43 (HCoV-OC43), severe acute respiratory syndrome coronavirus 2 (SARS-CoV-2)

## Abstract

Despite RT-PCR confirmed COVID-19, specific antibodies to SARS-CoV-2 spike are undetectable in serum in approximately 10% of convalescent patients after mild disease course. This raises the question of induction and persistence of SARS-CoV-2-reactive T cells in these convalescent individuals. Using flow cytometry, we assessed specific SARS-CoV-2 and human endemic coronaviruses (HCoV-229E, -OC43) reactive T cells after stimulation with spike and nucleocapsid peptide pools and analyzed cytokine polyfunctionality (IFN*γ*, TNFα, and IL-2) in seropositive and seronegative convalescent COVID-19 patients as well as in unexposed healthy controls. Stimulation with SARS-CoV-2 spike and nucleocapsid (NCAP) as well as HCoV spike peptide pools elicited a similar T cell response in seropositive and seronegative post COVID-19 patients. Significantly higher frequencies of polyfunctional cytokine nucleocapsid reactive CD4+ T cells (triple positive for IFN*γ*, TNFα, and IL-2) were observed in both, seropositive (p = 0.008) and seronegative (p = 0.04), COVID-19 convalescent compared to healthy controls and were detectable up to day 162 post RT-PCR positivity in seronegative convalescents. Our data indicate an important role of NCAP-specific T cells for viral control.

## Introduction

Coronavirus disease 2019 (COVID-19) is a respiratory disease caused by the newly emerged severe acute respiratory syndrome coronavirus 2 (SARS-CoV-2). In response to an infection with SARS-CoV-2, mechanisms of cellular and humoral immunity have been described (1–6). To date, it remains uncertain whether convalescent SARS-CoV-2 infected patients develop a robust and long lasting immunity and which parts of the cellular and humoral immune system are necessary for shaping this response.

Humoral immune response against SARS-CoV-2 is directed against spike (S) protein, as well as to other viral structural proteins, including membrane (M) and nucleocapsid protein (NCAP), with neutralizing antibodies mainly binding to the receptor binding domain (RBD) of the S trimer of SARS-CoV-2 (7, 8). Persistence of specific IgG antibodies to SARS-CoV-2 in serum correlates with clinical severity during acute infection and antibodies were found to decline more rapidly in asymptomatic patients (9). While high neutralizing antibody titer are only found in the minority of convalescent patients (7, 10), they were shown to correlate with numbers of virus-specific T cells (8, 11). Moreover, in convalescent patients, polyfunctional SARS-CoV-2-specific T cells have been described, indicating the generation of a memory-like phenotype (6, 12, 13).

Of note, approximately 10% of patients with a mild disease course do not show detectable IgG antibody levels to SARS-CoV-2 S protein in serum during the early convalescent phase (9, 10). This raises the question if SARS-CoV-2-reactive T cells are induced and persist in the seronegative patients.

When analyzing T cell responses to SARS-CoV-2, preexisting cross-reactive T cell responses to human endemic coronaviruses (HCoV) needs to be distinguished from those activated by SARS-CoV-2. Preexisting cross-reactive T cells to SARS-CoV-2 were described in 35–90% of unexposed healthy individuals (1–6). In a recent study we could show, that cross-reactive SARS-CoV-2 T cells in naïve patients can be distinguished from those post COVID-19 by a multifunctional cytokine secretion profile (IFNγ/IL-2/TNFα) after stimulation with peptide pools of SARS-CoV-2 (14).

In the present study, we comparatively analyzed SARS-CoV-2-reactive T cells in convalescent individuals with (seropositive) or without (seronegative) antibody response to SARS-CoV-2 S and NCAP peptide pools as well as to peptide pools of spike of HCoV-229E and HCoV-OC43. T cell reactivity, determined by activation markers CD137 and CD154 (CD40L), as well as cytokine secretion profiles (IFNγ/IL-2/TNFα) were assessed after stimulation with the corresponding peptide pools of SARS-CoV-2 and HCoV.

## Material and Methods

### Human Blood Samples

COVID-19 patients were diagnosed by RT-PCR and samples taken at least 4 weeks after positive RT-PCR. Convalescent patients were part of a cohort published previously (10). Healthy controls (HC) were recruited among laboratory staff, had no history of a COVID-19 episode and no SARS-CoV-2 detection, although unrestricted access to RT-PCR testing. Blood from post COVID-19 patients and HCs was collected from June to October 2020. The study was approved by the Ethics Committee of Charité Universitätsmedizin Berlin in accordance with the 1964 Declaration of Helsinki and its later amendments (EA2/092/20 from June 4th, 2020). All patients and controls gave informed consent.

### SARS-CoV-2 Serology

Serum IgA and IgG against the S1 domain of the SARS-CoV-2 Spike and the N domain of SARS-CoV-2 were determined by ELISAs according to the manufacturer’s instructions (Euroimmun Medizinische Labordiagnostika AG, Lübeck, Germany) and using the fully automated Euroimmun Analyzer I (Euroimmun Medizinische Labordiagnostika AG). Optical density (OD) ratios above 1.1 were considered reactive for both IgG and IgA. Furthermore, we applied, a solid phase immunoassay (SeraSpot^®^Anti-SARS-CoV-2 IgG, Seramun Diagnostica GmbH, Heidesee, Germany) based on the four recombinant SARS-CoV-2 proteins (complete Spike, S1 domain, RBD, and nucleocapsid protein). The SpotSight^®^plate scanner was used for measurements. Results are calculated and as normalized signal-to-cutoff (S/CO) ratios by dividing the observed signal strength of a specific spot by that of an internal cutoff control. Samples with an S/CO ratio of ≥1.0 were counted positive as defined by the manufacturer. Neutralizing IgG antibodies were determined by plaque reduction neutralization test (PRNT) similar as described before (15).

### Cell Isolation and Culture

Peripheral blood mononuclear cells (PBMCs) were isolated from heparinized whole blood by Biocoll density gradient centrifugation, frozen at −80°C and subsequently moved to liquid nitrogen. Cells from post COVID-19 patients with and without SARS-CoV-2 spike antibodies and HCs were analyzed simultaneously. Thawed PBMCs were rested for 24 h in Isocove Basal Medium with 10% fetal calf serum (FCS) and 1% penicillin/streptomycin (P/S) to exclude unspecific activation. Afterwards, 2 × 10^6^ PBMCs were stimulated with 1 µg/ml of peptide pools SARS-CoV-2 Spike Glycoprotein (two vials with N-term and C-Term, PM-WCPV-S-1), SARS-CoV-2 NCAP (PM-WCPV-NCAP-1), HCoV-229E Spike Glycoprotein (two vials with N-term and SII, PM-229E-S-1), and HCoV-OC43 Spike Glycoprotein S1 (two vials with N-term and C-term, JPT Peptide Technologies GmbH, Berlin), respectively. The background control was incubated with DMSO only. Stimulation with 3 µg/ml of superantigen Staphylococcal enterotoxin B (SEB) served as positive control to show that activation of T cells is not functionally impaired in any of the three groups analyzed. SEB is an extremely potent stimulant of T cells and activates ~20% of total T cells, whereas normal antigens activate only ~0.01% of total T cells (16). Samples were incubated for 18 h at 37°C and 5% CO_2_. After 2 h of stimulation, brefeldin A (BFA) was added as secretion inhibitor to the cell culture.

### Flow Cytometric Analysis of Antigen-Reactive T Cells

Peptide stimulated cells were washed and extracellularly stained with LIVE/DEAD Fixable Blue Dead Cell Stain Kit (Thermo Fisher Scientific). Afterwards, samples were fixed and permeabilized for 30 min at 4°C using FoxP3 transcription factor staining buffer set (eBioscience). Following, intracellular staining was performed for CD3 BV650, CD4 PerCp-Cy5.5, CD8 BV510, CD137 PE, CD154 BV421, IL-2 APC, IFNγ BV605, and TNFα AF700 (Biolegend). Stained cells were then transferred into a 96-well plate and measured at a CytoflexLX (Beckman Coulter). Flow cytometry data was analyzed using FlowJo software version 10.6.2 (BD). Reactive T cells were defined as CD154+CD137+CD4+ or CD137+CD8+ T cells >0.005% within total CD4+ or CD8+ T cells and with a threshold of ≥1.2-fold signal above the background control. This threshold corresponds to the range in which 95% of all negative samples are. Unspecific activation of cells was excluded by subtracting the background signal of the DMSO stimulated negative control sample from the peptide stimulated samples. Single, double (dp), or triple (tp) cytokine producing reactive T cell subsets were analyzed using Boolean combination gates (see **Supplementary Figure 1** for gating strategy).

### Statistical Analysis

Statistical analyses were performed using GraphPad Prism 6 software. Continuous variables were expressed as median and interquartile range (IQR). Univariate comparisons of T cell responses in two independent groups were done using non-parametric Mann-Whitney-U test.

A one-tailed p-value of <0.05 was considered statistically significant. Due to multiple testing p-values are considered descriptive.

## Results

### Patient Characteristics and Antibody Response

Of 206 convalescent individuals, 29 were tested negative for SARS-CoV-2 S-IgG, of which 23 convalescent individuals were seronegative for SARS-CoV-2 S-IgG and –IgA. After additional serological testing for NCAP, spike domains, RBD and S1, and full-spike IgG- and IgA-antibodies, we identified 21 patients that remained seronegative (see **Supplementary Table 1**). In this study eight of these seronegative (Ab−) convalescent individuals were analyzed for their T cell response and compared to seven seropositive convalescent individuals (Ab+) and to eight SARS-CoV-2 unexposed HCs (see **Supplementary Table 2**). The characteristics of post COVID-19 patients are shown in **Table 1**. All included post COVID-19 patients had a mild disease course (WHO R&D scale 2) and a median of 99 (Ab−) and 83 (Ab+) days after diagnosis before being analyzed for the T cell responses. All post COVID-19 (Ab+) patients had specific IgG and IgA as well as neutralizing IgG against SARS-CoV-2 (see **Supplementary Table 2**). HCs had no history of COVID-19. Median age for post COVID-19 Ab+ and Ab− was 39 (30–57) and 44 years (31–54) respectively. HCs had a median age of 38 years (27–57), all participants were Caucasian (see **Table 1**).

**Table 1:** Characteristics of seropositive and seronegative post COVID-19 patients.

ID	Age [years]	Sex	Time of serological analysis after diagnosis by pos. RT-PCR [d]	Time of T cell analysis after diagnosis by pos. RT-PCR [d]	Fever	Hospitalization due to COVID-19	WHO R&D Blueprint ordinal scale
**Seronegative (Ab−)**							
Ab− (#1)	50	m	37	77	yes	no	2
Ab− (#2)	38	m	53	88	no	no	2
Ab− (#3)	54	f	54	110	yes	no	2
Ab− (#4)	44	f	56	84	no	no	2
Ab− (#5)	41	m	33	69	no	no	2
Ab− (#6)	52	m	48	117	yes	no	2
Ab− (#7)	45	f	50	162	yes	no	2
Ab− (#8)	31	m	116	195	no	no	2
**Median**	44		51.5	99			
**Seropositive (Ab+)**							
Ab+ (#1)	34	w	50	198	yes	no	2
Ab+ (#2)	31	m	53	53	no	no	2
Ab+ (#3)	30	m	53	53	no	no	2
Ab+ (#4)	57	w	50	78	no	no	2
Ab+ (#5)	40	w	53	83	yes	no	2
Ab+ (#6)	55	m	43	89	yes	no	2
Ab+ (#7)	39	w	68	158	no	no	2
**Median**	39		50	83			

RT-PCR, reverse transcriptase polymerase chain reaction; f, female; m, male; d, days.

### Reactive T Cells in Response to SARS-CoV-2 and HCoV Peptides

In order to analyze T cell responses to SARS-CoV-2 and two common HCoV strains (HCoV-229E and -OC43) we used flow cytometry to first evaluate the frequencies of CD4+CD154+CD137+ and CD8+CD137+ T cells (for gating strategy see **Supplementary Figure 1**).

For CD4+CD154+CD137+ reactive T cells seven out of eight post COVID-19 Ab− had a T cell response to the spike N- and C-terminal domain of SARS-CoV-2. Five Ab− convalescent individuals also showed a robust T cell response to the SARS-CoV-2 NCAP peptide pool. In post COVID-19 Ab+ 7/7 had reactive T cells against S and 6/7 to NCAP of SARS-CoV-2. For HCs 6/8 participants had reactive T cells against S, whereas only four had a response to NCAP. All patients and controls with CD4+ responses to SARS-CoV-2 peptides also showed a response to at least one of the HCoV strains. Further, 6/8 post COVID-19 Ab− and 6/7 Ab+ had a CD8+ T cell response to S N- or C-terminal domain of SARS-CoV-2, whereas 6/8 of the post COVID-19 Ab− and 4/7 of the Ab+ had a CD8+ response to NCAP. Six of eight HC had reactive CD8+ T cells to either SARS-CoV-2 S or NCAP peptide pools.

**Figure 1:**
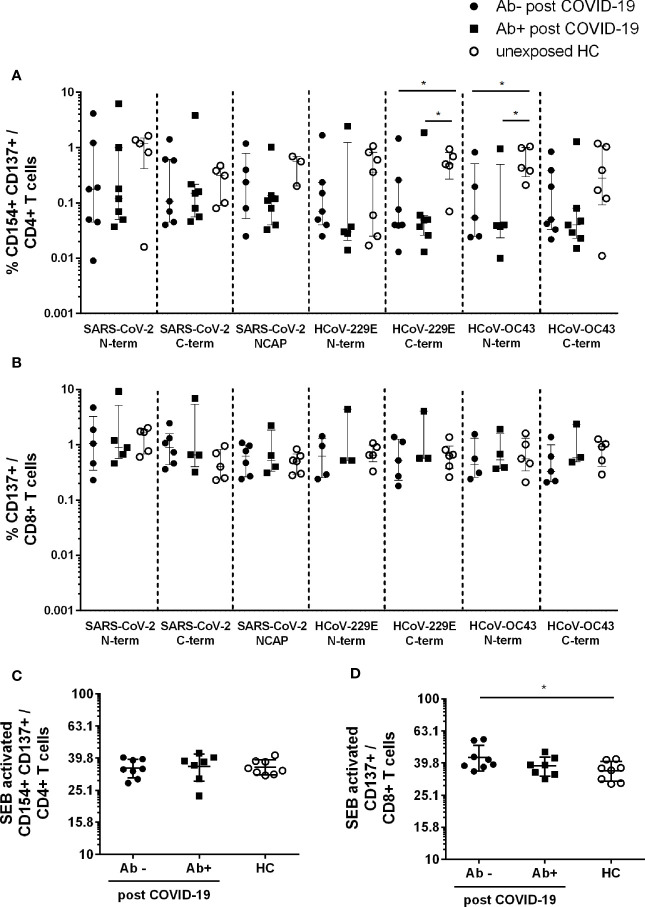
Activated CD4+ and CD8+ T cells in seropositive or –negative post COVID-19 in response to SARS-CoV-2 and HCoV peptide pools compared to HCs. Post COVID-19 Ab− (n = 7; filled black dot), post COVID-19 Ab+ (n = 8; filled black squares), and HC (n = 8; empty black dots) were analyzed. Frequencies of activated CD4+CD154+CD137+ **(A)** and CD8+CD137+ **(B)** T cells after SARS-CoV-2 and HCoV peptide stimulation. Frequencies of activated CD4+ **(C)** and CD8+ **(D)** T cells after stimulation with SEB. Only T cell responses above the threshold of 20% above background activation are shown. Median and interquartile range (IQR) are indicated. Statistical analysis was performed by non-parametric one-tailed Mann–Whitney-U test for comparison of control and patient groups. A p-value ≤0.05 was considered as statistically significant. *p ≤ 0.05.

Frequencies of reactive CD4+ and CD8+ T cells were comparable among the three groups in response to SARS-CoV-2 peptides (**Figures 1A, B**), whereas HC had significantly higher frequencies of reactive CD4+ T cells in response to HCoV-229E S C-term (Ab−: p = 0,05; Ab+ p = 0.02) and HCoV-OC43 S N-term (Ab−: p = 0.03; Ab+: p = 0.03) compared to post COVID-19 (**Figure 1A**). No difference in response to HCoV was observed in activated CD8+ T cells (**Figure 1B**). Moreover, similar frequencies of CD4+ and CD8+ activated T cells in response to SEB were observed (**Figures 1C, D**). Post COVID-19 seronegative patients had slightly higher levels of SEB activated CD8+ T cells compared to HC (**Figure 1D**).

### Cytokine Profile of SARS-CoV-2 and HCoV Reactive T Cells

**Figure 2:**
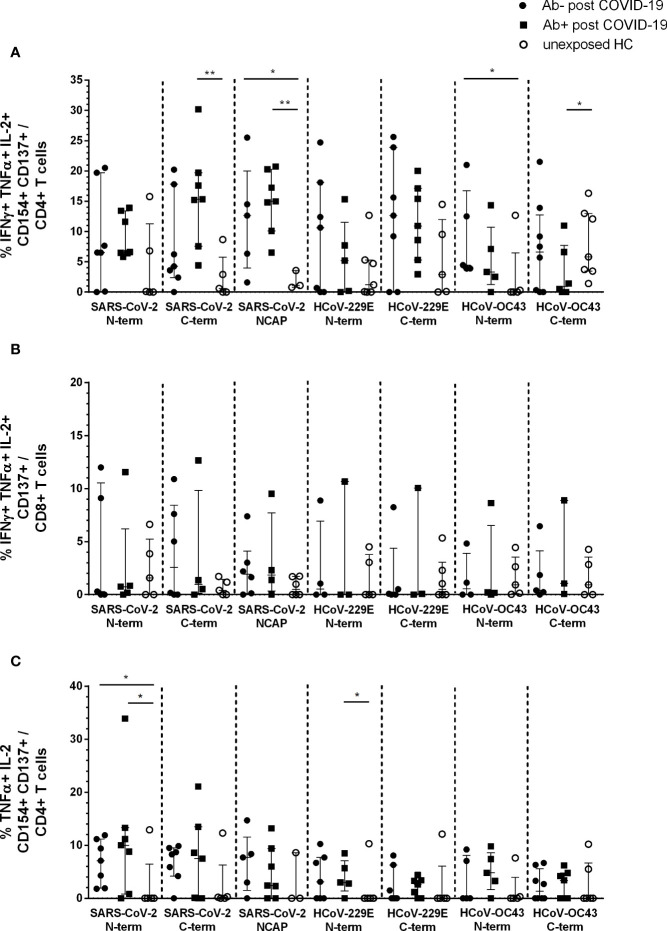
Triple and double cytokine producing activated CD4+ and CD8+ T cells in seropositive and -negative post COVID-19 in response to SARS-CoV-2 and HCoV peptide pools compared to HCs. IFNγ, TNFα, and IL-2 triple producing activated CD4+ and CD8+ T cells were analyzed by Boolean combination gating strategy. Cytokine expression profile in triple producing activated CD4+CD154+CD137+ **(A)** and CD8+CD137+ **(B)** T cells, as well as TNFα and IL-2 double producing activated CD4+ T cells **(C)** in response to SARS-CoV-2 and HCoV peptide pools are shown. Median and interquartile range (IQR) are indicated. Statistical analysis was performed by non-parametric one-tailed Mann–Whitney-U test for comparison of control and patient groups. A p-value ≤0.05 was considered as statistically significant. *p ≤ 0. 05; **p ≤ 0.001.

Using Boolean combination gating, the percentage of cytokine producing activated CD4+ and CD8+ T cells was analyzed. Single (sp) (**Supplementary Figures 2A** and **3A**), double (dp) (**Supplementary Figures 2B** and **3B**), and triple (tp) (**Figure 2**) cytokine producing CD4+CD154+CD137+ and CD8+CD137+ T cells were depicted. The highest number of cytokine-reactive T cells in response to the seven peptide pools were IFNγ+ TNFα+ IL-2+ tp activated CD4+ and CD8+ T cells (**Figures 2A, B**) as well as TNFα+ IL-2+ dp CD4+ (**Figure 2C**) and IFNγ+ TNFα+ CD8+ (**Supplementary Figures 3Ba**) reactive T cells.

The main difference in post COVID-19 *versus* unexposed HC were observed in IFNγ+TNFα+IL-2+ tp activated CD4+ T cells. Post COVID-19 Ab+ had significantly higher activated CD4+ T cells in response to SARS-CoV-2 S C-terminal peptide pool compared to HC (p = 0.005). Both, post COVID-19 Ab− and Ab+, had significantly higher cytokine tp CD4+ NCAP reactive T cells compared to HC (Ab−: p = 0.04; Ab+: p = 0.008). Moreover, post COVID-19 Ab− had significantly higher tp HCoV-OC43 S N-terminal reactive T cells (p = 0.05) and post COVID-19 Ab+ less tp HCoV-OC43 S C-terminal reactive CD4+ T cells (**Figure 2A**). No difference in tp activated CD8+ T cells was observed (**Figure 2B**).

Both post COVID-19 groups had significantly higher dp TNFα+ IL-2+ SARS-CoV-2 S N-term reactive CD4+ T cells compared to HC (Ab−: p = 0.04; Ab+ p = 0.05). Further, post COVID-19 Ab+ had significantly higher HCoV-229E S N-term reactive CD4+ T cells (p = 0.05) (**Figure 2C**).

The difference in tp CD4+ NCAP reactive T cells was even more prominent, when we pooled the data of this study with HCs and post COVID-19 Ab+ of our already published cohort (14) (**Supplementary Figure 4**). Post COVID-19 Ab− (p = 0.018) and Ab+ (p = >0.0001) had significantly higher frequencies of tp activated CD4+ T cells compared to HC (**Supplementary Figure 4C**). Strikingly, when pooling the data this difference shows in CD8+ T cells as well (post COVID-19 Ab+: p = 0.05; post COVID-19 Ab−: p = 0.02; **Supplementary Figure 4D**). Moreover, we observed significantly higher frequencies of tp SARS-CoV-2 S C-terminal reactive CD4+ T cells in post COVID-19 Ab+ compared to HC (p = 0.0008, **Supplementary Figure 4C**). Again, frequency of activated CD4+ or CD8+ T cells did not differ significantly between the three groups (**Supplementary Figures 4A, B**).

In the seronegative convalescent group, tp reactive CD4+ cells to NCAP were detectable up to day 162 after positive RT-PCR. In this study, latest time point for triple positive CD4+ cell reactivity to SARS-CoV-2 C-terminal spike (S2) in a seronegative convalescent individual was at day 195 after RT-PCR confirmed infection.

Taken together, when analyzing SARS-CoV-2 S- and NCAP-specific T cell response, no difference between T cells expressing activation markers CD154 and CD137 were found between post COVID-19 and unexposed HC. However, both S and NCAP-specific tp and S dp producing CD4+ T cells were significantly increased in seropositive and seronegative post COVID-19 patients compared to unexposed HC, providing evidence for specific T cell responses in Ab− post COVID-19.

## Discussion

In mild COVID-19, specific IgG antibodies to SARS-CoV-2 are undetectable in approximately 10% of convalescent individuals (9, 10). In addition, there are reports on patients with immune deficiencies that suffer only mild COVID-19 disease despite their inability to mount specific antibodies against SARS-CoV-2 (17–24) In this study, we provide evidence that SARS-CoV-2-reactive T cells to S and NCAP of SARS-CoV-2 can be identified by dp spike and tp NCAP profiles in seronegative patients with mild COVID-19.

The applied flow cytometry based approach used in our study enables to discriminate different reactive T cell populations. In addition, flow cytometry allows for the evaluation of polyfunctionality of reactive T cells by measuring multiple cytokine secretion patters (sp, dp, or tp IFNγ, TNFα, and IL-2) instead of IFNγ secretion alone. By stimulating with S peptide pools of HCoV-229E and -OC43, our flow cytometric approach allows to put the T cell reactivity into perspective with possible cross-reactivities of SARS-CoV-2. There is evidence, that ~90% of the population worldwide express IgG seropositivity to the circulating endemic HCoV strains (25). In our previous study we observed a high correlation of T cells reactive against spike N- or C-terminus of HCoV and SARS-CoV-2 in unexposed but not post COVID-19 HC suggesting a cross-reactivity of pre-existing T cells (14). This finding is in line with studies from Nelde et al. and Mateus et al., providing evidence for homology of many MHC epitopes of the spike protein between HCoV and SARS-CoV-2 (4, 5). This presence of cross-reactive T cells to various peptide pools of SARS-CoV-2 in unexposed healthy individuals has been reported by various groups ranging from 35 to 90% (1–6). These differences likely depend on the sensitivity of different assays, the type of peptide pools being used and the time of analysis.

Frequencies of CD154+ CD137+ reactive T cells were similar in seronegative and seropositive convalescent individuals. T cell reactivity in response to S peptide pools of HCoVs were variable with slightly lower frequencies of CD4+CD154+CD137+ activated T cells reactive to C-terminal S of HCoV-229E and -OC43 in post COVID-19 individuals compared to HC.

Triple positive (IFN*γ*+/IL-2+/TNFα+) CD4+ T cells responsive to NCAP peptide pool, indicating a robust T cell response, do not differ between Ab− and Ab+ and are significantly higher in all convalescent individuals (seropositive and seronegative) compared to unexposed HCs. In a pooled data analysis, including our previously published convalescent and unexposed HCs (14), this difference is also of statistical significance for NCAP-reactive tp CD8+ T cells (see **Supplementary Figure 1**).

Looking at tp (IFN*γ*+/IL-2+/TNFα+) CD4+ reactive T cells in response to spike peptide pools of HCoV, seropositive and seronegative convalescent individuals showed no difference. In addition, longevity of T cell responses are similar. Median day of analysis was at day 83 (Ab+) *vs* day 99 (Ab−) after positive RT-PCR, with polyfunctional tp CD4+ cells to NCAP and spike being detectable at least until day 162 and day 195 after positive RT-PCR testing in seropositive and seronegative convalescents respectively. Schwarzkopf et al. recently also detected a T cell response against SARS-CoV-2 M and S protein in 7/9 seronegative convalescent COVID-19 individuals by using an in-house IFN*γ* ELISpot assay (26).

Without focusing on seronegative convalescent patients, polyfunctional SARS-CoV-2 reactive T-cells, indicating a memory-like phenotype, were previously described in mild convalescent patients during early convalescent period (12, 27). Our cohort enables to study the SARS-CoV-2 T cell response in the absence of detectable IgG- and IgA-antibodies in serum and expands our knowledge on the longevity of T cell response by detecting reactive T cells for up to 195 days post symptom onset. Different time points of T cell analysis and mild disease course might also explain the lower expression of IL-2 in our patients (28, 29).

Although the potential protective role of pre-existing SARS-CoV-2 T cells remains to be clarified in prospective studies, in this study seronegative and seropositive convalescent individuals express similar frequencies of reactive T cells to S peptide pools of HCoVs. Therefore, at least in our cohort, a lack of specific antibody response does not correlate with differences in reactive T cells to HCoVs, arguing against a role in facilitating the mounting of SARS-CoV-2 antibody responses.

Overall, our findings provide evidence that seronegative convalescent individuals have mounted a SARS-CoV-2 T cell response comparable to seropositive.

Humoral immune responses occur at different compartments and the absence of anti-SARS-CoV-2-specific antibodies in the peripheral blood does not rule out the presence of reactive antibodies at mucosal sites. In the present study, mucosal antibodies were not analyzed, hence we cannot address its effects on the immune response to COVID-19. Although dimeric IgA antibodies were shown to enhance SARS-CoV-2 neutralization, the overall role of specific IgA remains to be determined (30). Comparative data for serum and mucosal antibodies are limited, however similarities between levels of blood and saliva IgG were observed (31). Also Cervia et al. showed that mucosal SARS-CoV-2 spike protein-specific IgG levels are related to systemic titers. However, in their cohort, approximately 20% of convalescent patients negative for SARS-CoV-2 IgG had nevertheless specific antibodies detected in mucosal fluids (32). It is intriguing to speculate, that preformed cross-reactive mucosal antibodies could form an effective first line of defense against SARS-CoV-2. While this hypothesis remains to be tested in prospective studies, our data indicate that a memory-like T-cell response to NCAP is generated irrespective of an antibody formation in serum. It is expected, that evaluating the clinical course of COVID-19 in patients suffering from selective IgA deficiency could contribute to our understanding of mucosal immunity (33). Due to very low case numbers and different reports on clinical outcome, effects of antibody deficiency remain inconclusive (19–22). However, considering the relatively high number of recovered patients with X-linked agammaglobulinemia (XLA), it is becoming clear, that other factors than specific antibodies to SARS-CoV-2 are contributing to clinical resolution. In the present study, all convalescent individuals with undetectable SARS-CoV-2 antibodies in serum had mild disease courses, also indicating a robust and sufficient immune response.

CD4+ T cells play a vital role in the control of many viral infections by generating neutralizing antibodies and priming of CD8+ T cells (34–37). Effective T cell-mediated control of viral infections is characterized by the production of different cytokines, such as IFNγ, IL-2, and TNFα (38–41). The co-expression of T cell activation markers, e.g. CD154 (CD40L), together with polyfunctional cytokine secretion profiles were shown to be associated with enhanced viral control (42). CD154 and CD137 are highly reliable functional markers allowing a comprehensive characterization of the total pool of antigen-specific T cells irrespective of functional specialization and are upregulated within the 16h of stimulation (43).

Our study has important limitations. Although cytokine polyfunctionality and activation markers add further information on the nature of SARS-CoV-2 T cell response in comparison to IFNγ ELISpot assays alone, it would be of great interest to characterize T cell subsets with a more detailed marker profile e.g. for T helper cell subsets and to expand to other activation markers, such as Ki67, CD38, CD69, or CD107a (12).

Moreover, our sample size is limited due to the relatively low percentage of patients lacking an antibody response after SARS-CoV-2 infection and persistence of tp reactive T cells at later time points remain to be determined. Furthermore, our study focused on the humoral and T cell response to S and NCAP protein only. Future studies would benefit from even broader approaches by including other SARS-CoV-2 protein regions. In addition, assessing mucosal SARS-CoV-2 antibodies and a more detailed virological data (e.g. viral load) would certainly help to augment our understanding of SARS-CoV-2 immune responses. Most importantly, prospective cohort studies are required in order to address the potential protective role of (cross) reactive T cells and antibodies in different compartments.

Our finding of tp T cells reactive to SARS-CoV-2 NCAP indicate an important role of the T cellular immune response to this viral protein. Current vaccines focus on the generation of S antibodies (44). It is unknown, if or to what extent, a mere T cell response to SARS-CoV-2 S protein could provide protection in patients who lost or are unable to mount a humoral immune response. Future studies should address, too, if a vaccine-induced immune response to other viral protein structures (e.g. to NCAP) might confer even higher rates of protection from disease and viral transmission in patients with impaired humoral immune responses, such as in patients after chemotherapy, B cell depletion, or in primary immunodeficiencies, who were shown be at higher risk for severe clinical COVID-19 disease (23, 45).

## Data Availability Statement

The raw data supporting the conclusions of this manuscript will be made available by the authors, without undue reservation.

## Ethics Statement

The studies involving human participants were reviewed and approved by the Ethics Committee of Charité Universitätsmedizin Berlin in accordance with the 1964 Declaration of Helsinki and its later amendments (EA2/092/20 from June 4th, 2020). The patients/participants provided their written informed consent to participate in this study.

## Author Contributions

CS and LH made substantial contributions to conception and design. LH made patient samples available. SS and SB performed acquisition and analysis of data. SS, FS, LH, and CS performed interpretation of data. CD, VC, and TS performed analysis of antibody titers. LH and SS wrote the article. H-DV, CD, VC, TS, FS, and CS reviewed the manuscript critically for important intellectual content. All authors contributed to the article and approved the submitted version.

## Funding

This research did not receive any specific grant from funding agencies in the public, commercial, or not-for-profit sectors. We acknowledge support from the German Research Foundation (DFG) and the Open Access Publication Fund of Charité - Universitätsmedizin Berlin.

## Conflict of Interest

VC is named together with Euroimmun on a patent application filed recently regarding detection of antibodies against SARS-CoV-2.

The remaining authors declare that the research was conducted in the absence of any commercial or financial relationships that could be construed as a potential conflict of interest.
