# Supplementary material for: Reactive T Cells in Convalescent COVID-19 Patients With Negative SARS-CoV-2 Antibody Serology
Source: Front Immunol. 2021 Jul 12;12:687449. doi: 10.3389/fimmu.2021.687449 (PMC8312095; doi:10.3389/fimmu.2021.687449)
Supplement: Supplementary file 1 [file DataSheet_1.docx]

Supplementary Material

# Supplementary Figures and Tables

Supplementary tables can found as Word files under the following names:

**Supplementary Table 1:** Detailed serological data of all convalescent individuals seronegative for spike-IgG by EUROIMMUN screening**.**

**Supplementary Table 2:** Detailed serological data of all seropositive convalescent individuals and healthy controls.

## Supplementary Figures


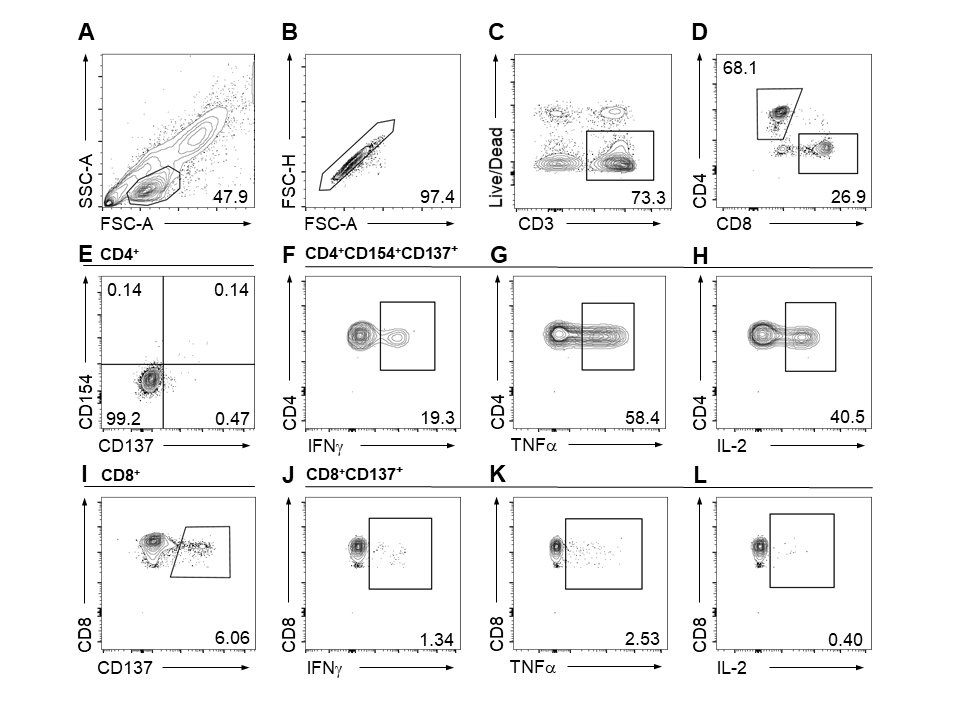


**Supplementary Figure 1: Gating Strategy for flow cytometry analysis of peptide reactive CD4+ and CD8+ T Cells and expression of cytokines in response to stimulation with SARS-CoV-2 S C-terminal peptide pool exemplarily shown in a seropositive convalescent COVID-19 patient.**  Depicted are lymphocytes **(A)**, single lymphocytes **(B)**, living CD3+ T cells **(C)**, CD4+ and CD8+ T cells **(D)**, activated CD4+CD154+CD137+ T cells **(E)** and the corresponding expression of the cytokines IFNγ, TNFα and IL-2 **(F-H)**, as well as activated CD8+CD137+ T cells **(I)** and the corresponding expression of the cytokines IFNγ, TNFα and IL-2 **(J-K)**. Single, double or triple cytokine producing activated T cell subsets were analyzed by Boolean combination gating using FlowJo software.


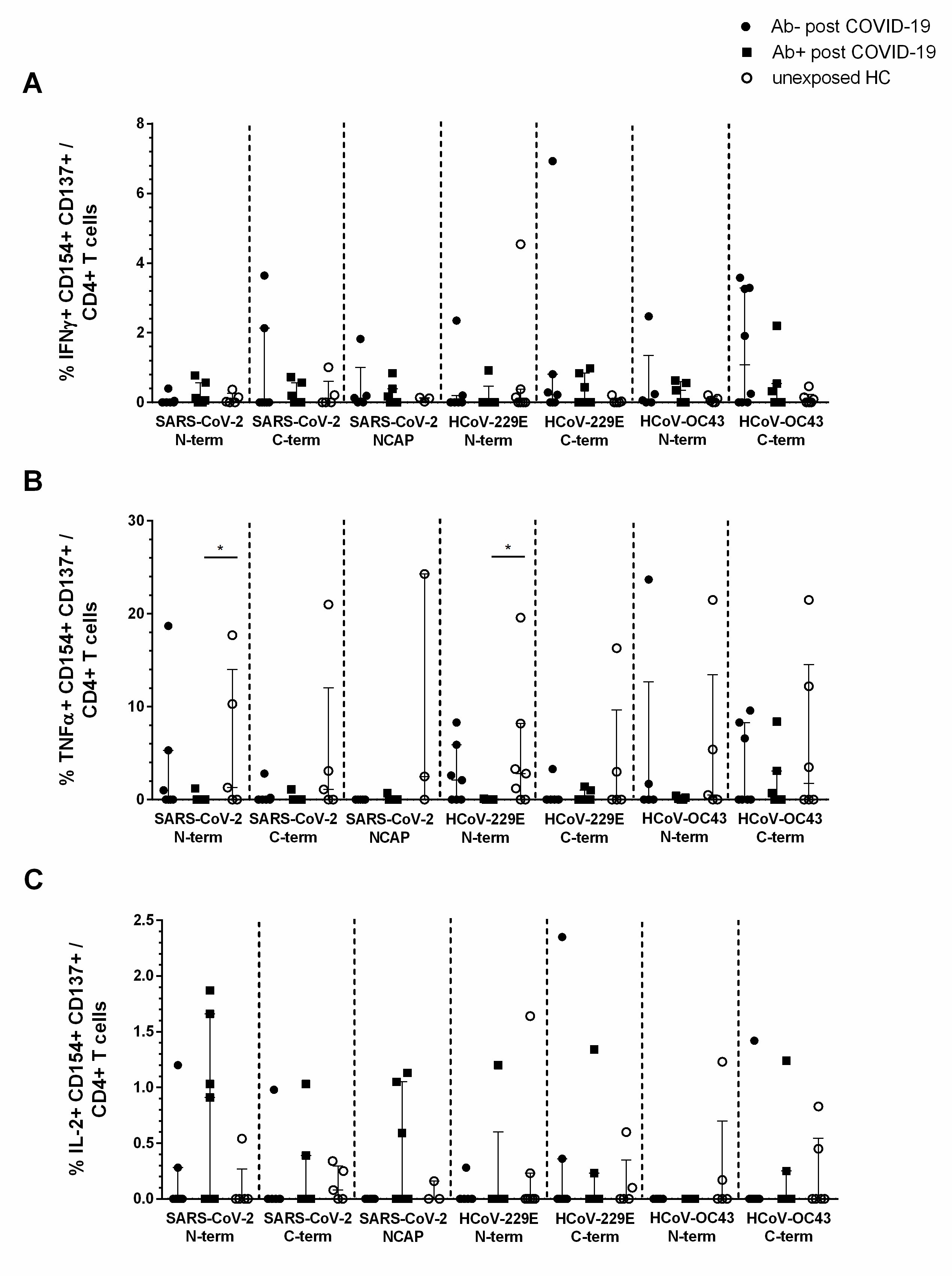


**Supplementary Figure 2a: Single cytokine producing activated CD4+ T cells in seropositive and -negative post COVID-19 in response to SARS-CoV-2 and HCoV peptide pools compared to HCs**. IFNγ, TNFα, or IL2 single producing (sp) activated CD4+ T cells were analyzed by Boolean combination gating strategy. IFNγ (A), TNFα (B) and IL2 (C) sp activated CD4+ T cells in response to peptide pools (1µg/ml) are shown. Median and interquartile range (IQR) are indicated. Statistical analysis was performed by non-parametric two-tailed Mann–Whitney-U test for comparison of control and patient groups. A p-value ≤ 0.05 was considered as statistically significant. p ≤ 0.05 = *; p ≤ 0.001 = **


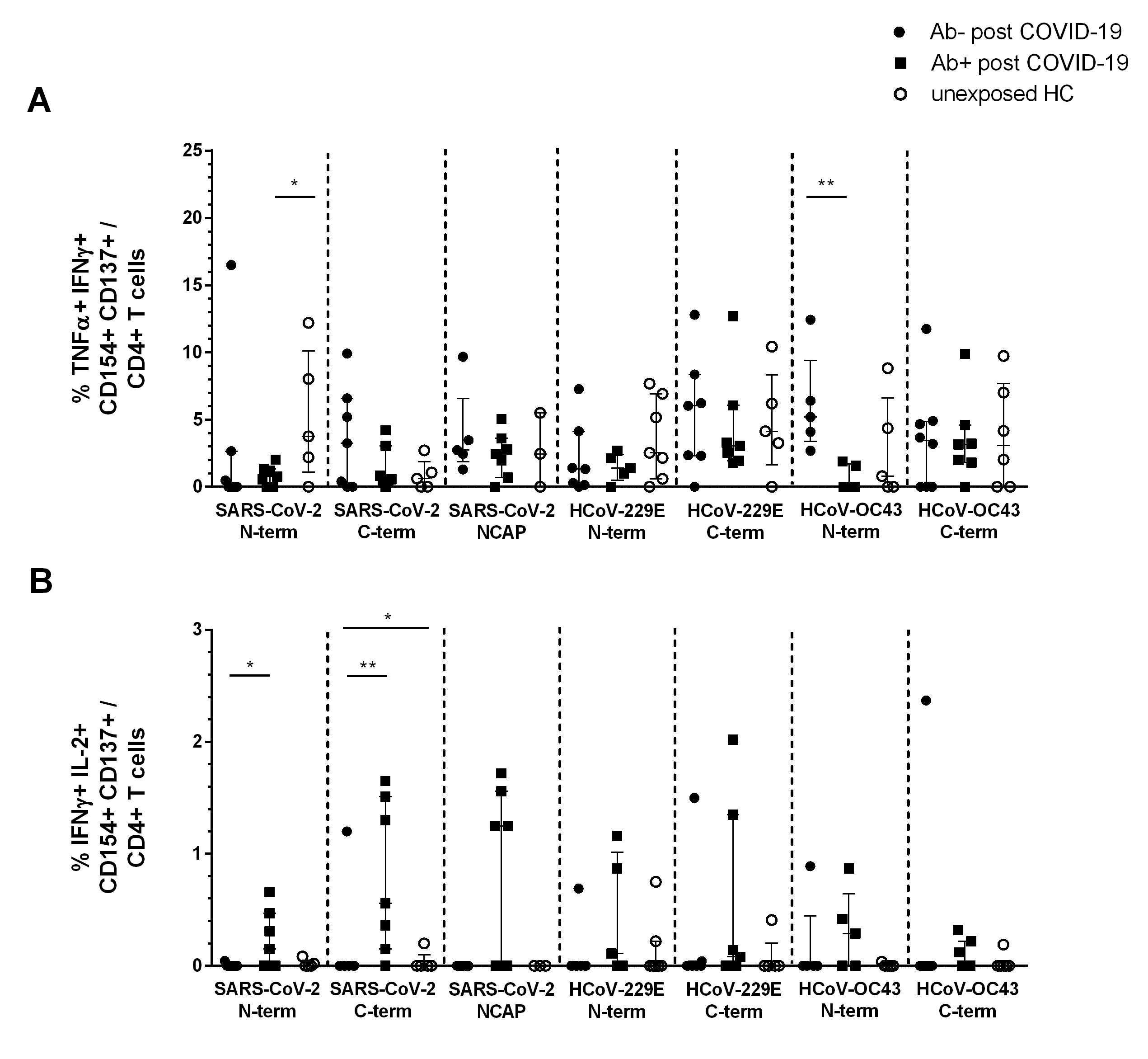


**Supplementary Figure 2b: Double cytokine producing activated CD4+ T cells in seropositive and -negative post COVID-19 in response to SARS-CoV-2 and HCoV peptide pools compared to HCs**. IFNγ, TNFα, or IL2 double producing (dp) activated CD4+ T cells were analyzed by Boolean combination gating strategy. TNFα/IFNγ (A) and IFNγ/IL2 (B) dp activated CD4+ T cells in response to peptide pools (1 µg/ml) are shown. Median and interquartile range (IQR) are indicated. Statistical analysis was performed by non-parametric two-tailed Mann–Whitney-U test for comparison of control and patient groups. A p-value ≤ 0.05 was considered as statistically significant. p ≤ 0.05 = *; p ≤ 0.001 = **


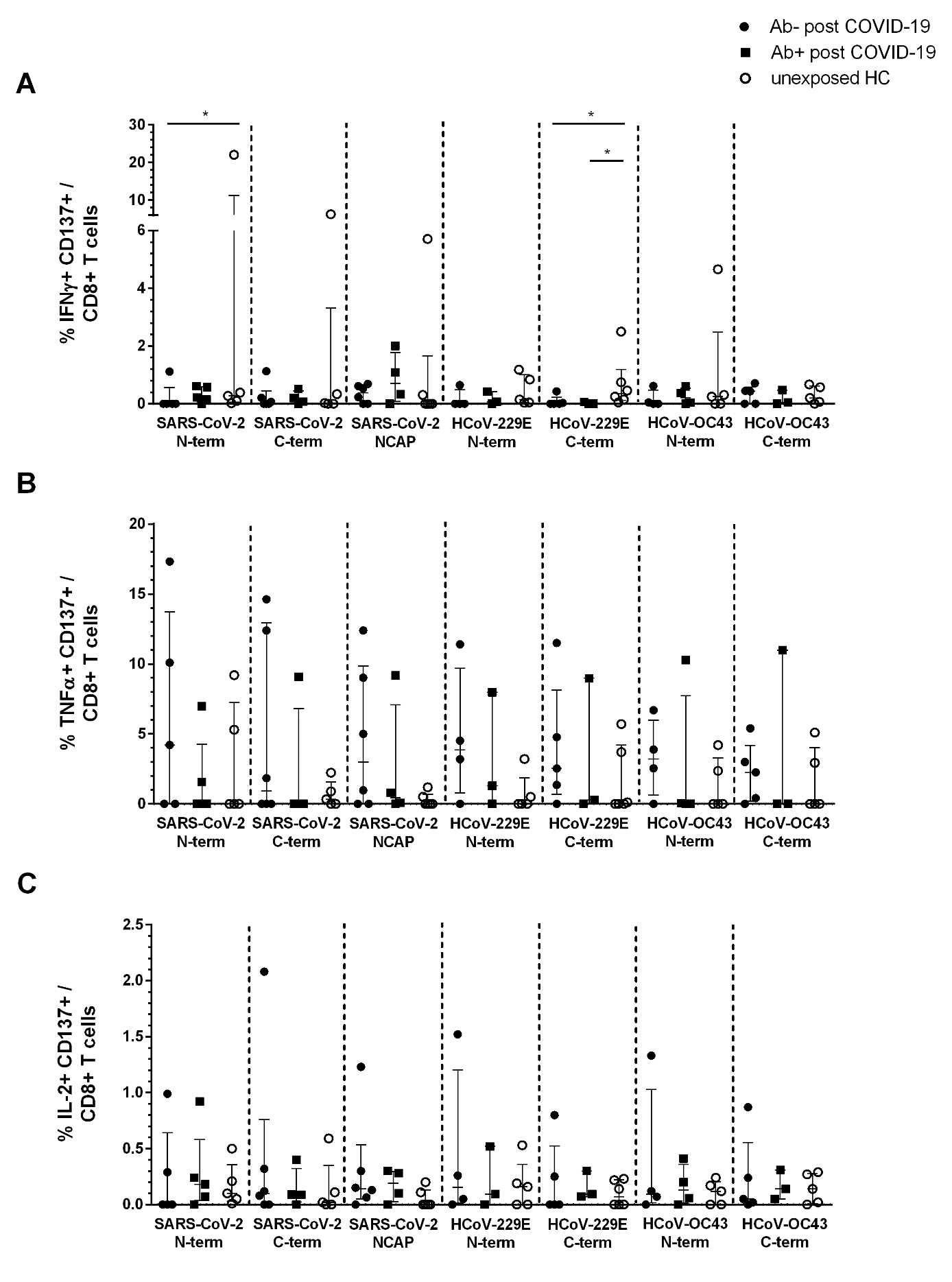


**Supplementary Figure 3a: Single cytokine producing activated CD8+ T cells in seropositive and -negative post COVID-19 in response to SARS-CoV-2 and HCoV peptide pools compared to HCs**. IFNγ, TNFα, or IL2 single producing (sp) activated CD8+ T cells were analyzed by Boolean combination gating strategy. IFNγ (A), TNFα (B) and IL2 (C) sp activated CD8+ T cells in response to peptide pools (1 µg/ml) are shown. Median and interquartile range (IQR) are indicated. Statistical analysis was performed by non-parametric one-tailed Mann–Whitney-U test for comparison of control and patient groups. A p-value ≤ 0.05 was considered as statistically significant. p ≤ 0.05 = *; p ≤ 0.001 = **


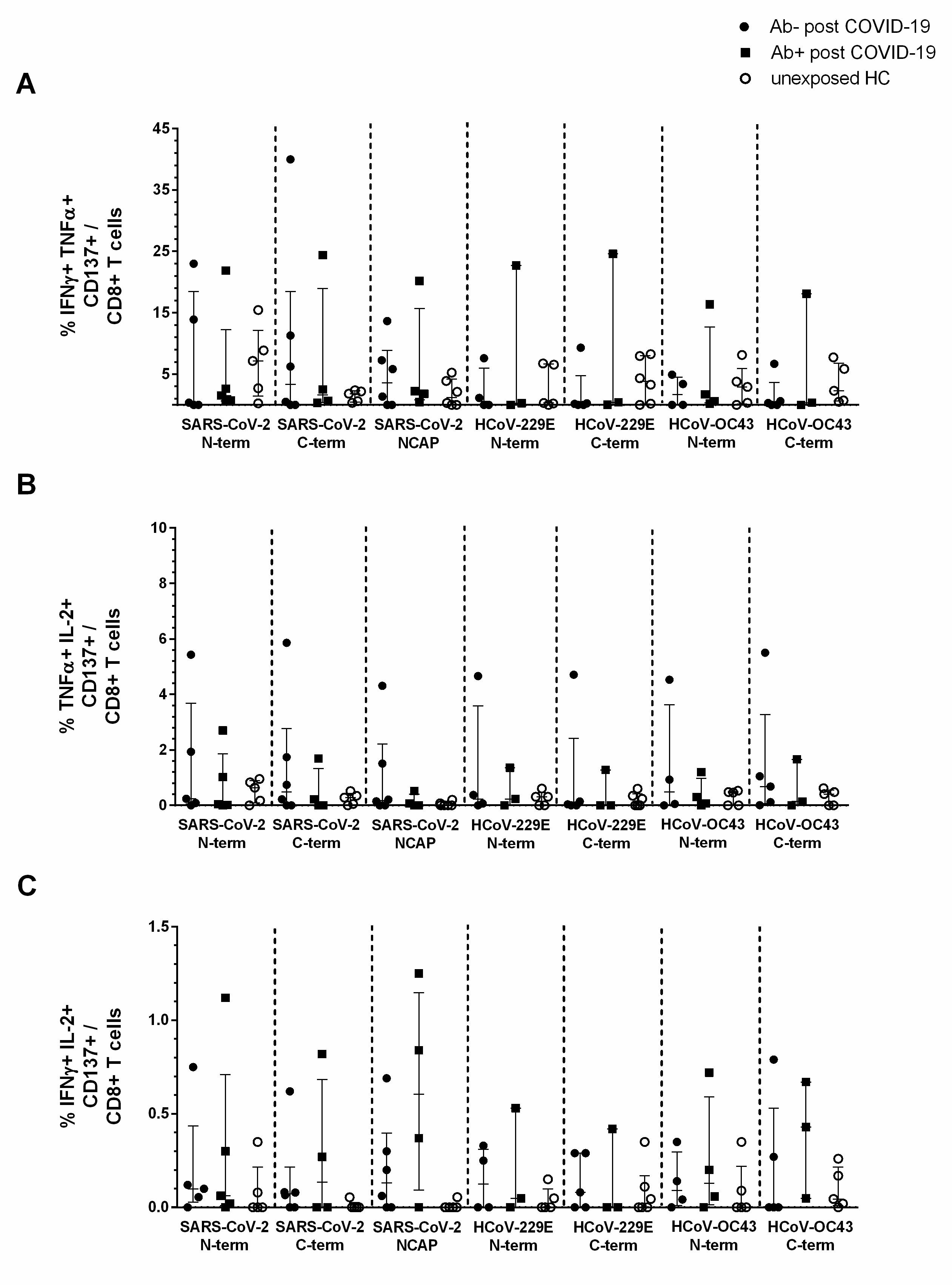


**Supplementary Figure 3b: Single cytokine producing activated CD8+ T cells in seropositive and -negative post COVID-19 in response to SARS-CoV-2 and HCoV peptide pools compared to HCs.** IFNγ, TNFα, or IL2 double producing (dp) activated CD8+ T cells were analyzed by Boolean combination gating strategy. IFNγ/TNFα (A), TNFα/IL-2 (B) and IFNγ/IL2 (C) dp activated CD8+ T cells in response to peptide pools (1 µg/ml) are shown. Median and interquartile range (IQR) are indicated. Statistical analysis was performed by non-parametric one-tailed Mann–Whitney-U test for comparison of control and patient groups. A p-value ≤ 0.05 was considered as statistically significant. p ≤ 0.05 = *; p ≤ 0.001 = **


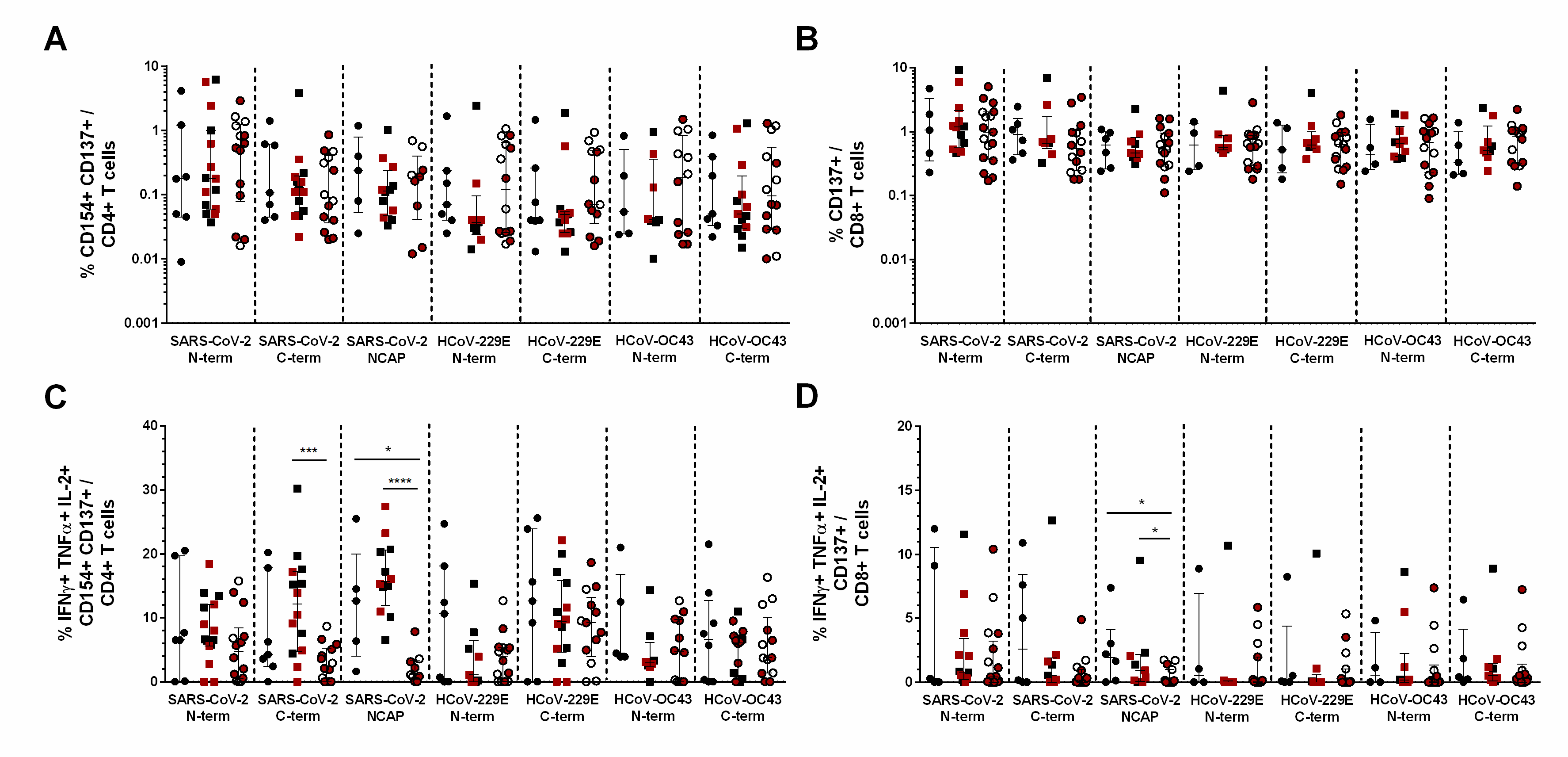


**Supplementary Figure 4: Pooled data analysis of activated CD4+ and CD8+ T cell frequencies and triple cytokine producing activated T cells in seropositive and –negative post COVID-19 in response to the peptide pools compared to HCs.** PBMCs of post COVID-19 Ab- (n=8; filled black dot), post COVID-19 Ab+ (n=7; filled black squares), HC (n=8; empty black dots) of this study and PBMCs of post COVID-19 Ab+ (n=12; filled red squares) and HC (n=12; filled red dots) of the former study (14) were pooled for analysis. Frequencies of activated CD4+ **(A)** and CD8+ **(B)** T cells after peptide stimulation. Frequencies of activated IFNγ, TNFα and IL-2 triple cytokine producing CD4+ **(C)** and CD8+ **(D)** T cells. Triple producing activated CD4+ and CD8+ T cells were analyzed by Boolean combination gating strategy. Median and interquartile range (IQR) are indicated. Statistical analysis was performed by non-parametric two-tailed Mann–Whitney-U test for comparison of control and patient groups. A p-value ≤ 0.05 was considered as statistically significant. p ≤ 0.05 = *; p ≤ 0.001 = **; p ≤ 0.0001 = ***, p ≤ 0.00001 = ****
