# Supplementary material for: Reactive T Cells in Convalescent COVID-19 Patients With Negative SARS-CoV-2 Antibody Serology
Source: Front Immunol. 2021 Jul 12;12:687449. doi: 10.3389/fimmu.2021.687449 (PMC8312095; doi:10.3389/fimmu.2021.687449)
Supplement: Supplementary file 2 [file Table_1.docx]

**Supplementary Table 1:** Detailed serological data of all convalescent individuals seronegative for spike-IgG by EUROIMMUN screening.

|  | **EUROIMMUN** | | **IgG** | | | | | **IgA** | | | | |  | |
| --- | --- | --- | --- | --- | --- | --- | --- | --- | --- | --- | --- | --- | --- | --- |
|  | **S-IgG** | **S-IgA** | **NCAP** | **RBD** | **S1** | **full spike** | **result** | **NCAP** | **RBD** | **S1** | **full spike** | **result** | **PRNT50** |  |
| **Ab- (1)** | **0.4** | **1.32** | **0.8** | **0.16** | **0.12** | **0.18** | **negative** | **0.11** | **0.11** | **0.04** | **0.09** | **negative** | **0** |  |
| **Ab- (2)** | **0.08** | **0.29** | **0.1** | **0** | **0** | **0** | **negative** | **0.09** | **0.04** | **0** | **0** | **negative** | **0** |  |
| **Ab- (3)** | **0.54** | **0.39** | **0.95** | **0.7** | **0.05** | **0.19** | **negative** | **0.13** | **0.05** | **0.03** | **0** | **negative** | **0** |  |
| **Ab- (4)** | **0.69** | **0.28** | **0.61** | **0.31** | **0.1** | **0.16** | **negative** | **0** | **0** | **0** | **0** | **negative** | **<20** |  |
| **Ab- (5)** | **0.09** | **0.2** | **0.03** | **0** | **0** | **0** | **negative** | **0.11** | **0** | **0.13** | **0** | **negative** | **0** |  |
| **Ab- (6)** | **0.18** | **0.21** | **0.05** | **0** | **0** | **0** | **negative** | **0** | **0** | **0** | **0** | **negative** | **0** |  |
| **Ab- (7)** | **0.55** | **0.47** | **0.75** | **0.28** | **0.06** | **0.16** | **negative** | **0.03** | **0** | **0** | **0** | **negative** | **0** |  |
| **Ab- (8)** | **0.22** | **0.32** | **0.33** | **0** | **0.02** | **0.02** | **negative** | **0** | **0** | **0** | **0** | **negative** | **0** |  |
| **Case 9** | 1.02 | 0.46 | 0.51 | 1.05 | 0.21 | 1.14 | positive | 0 | 0.05 | 1.12 | 0.05 | positive | 0 |  |
| **Case 10** | 0.81 | 0.74 | 1.59 | 0.54 | 0.17 | 0.26 | positive | 0.08 | 0 | 0 | 0 | negative | 20 |  |
| **Case 11** | 0.12 | 0.15 | 0.07 | 0.05 | 0 | 0 | negative | 0.02 | 0.02 | 0 | 0.02 | negative | 0 |  |
| **Case 12** | 0.17 | 0.27 | 0.22 | 0 | 0.25 | 0 | negative | 0 | 0 | 0.18 | 0 | negative | 0 |  |
| **Case 13** | 0.91 | 3.08 | 1.18 | 0.79 | 0.33 | 0.38 | positive | 0.63 | 0.69 | 0.11 | 0.29 | negative | 40 |  |
| **Case 14** | 0.61 | 0.86 | 0.91 | 0.51 | 0.12 | 0.26 | negative | 0.02 | 0.07 | 0 | 0 | negative | 0 |  |
| **Case 15** | 0.4 | 0.48 | 1.43 | 0.59 | 0.09 | 0.27 | positive | 0 | 0.09 | 0 | 0.03 | negative | 0 |  |
| **Case 16** | 0.14 | 0.84 | 0.11 | 0.17 | 0.02 | 0.37 | negative | 0 | 0.13 | 0 | 0.05 | negative | 0 |  |
| **Case 17** | 0.95 | 0.68 | 2.98 | 1.28 | 0.47 | 0.65 | positive | 0.22 | 0.06 | 0.03 | 0.03 | negative | 80 |  |
| **Case 18** | 0.69 | 0.86 | 0.23 | 0.64 | 0.18 | 0.2 | negative | 0 | 0.06 | 0.06 | 0.25 | negative | 0 |  |
| **Case 19** | 0.21 | 1.83 | 0.39 | 0.05 | 0.04 | 0.02 | negative | 0.2 | 0.02 | 0.04 | 0 | negative | 0 |  |
| **Case 20** | 0.33 | 0.27 | 0.62 | 0.4 | 0 | 0.19 | negative | 0.05 | 0 | 0 | 0 | negative | 0 |  |
| **Case 21** | 0.83 | 1.45 | 1.46 | 0.9 | 0.29 | 0.56 | positive | 0.09 | 0.12 | 0.02 | 0.12 | negative | 20 |  |
| **Case 22** | 1.05 | 1.05 | 0.55 | 0.65 | 0.2 | 0.45 | negative | 0.02 | 0.05 | 0 | 0 | negative | 0 |  |
| **Case 23** | 0.26 | 0.07 | 0.1 | 0 | 0 | 0 | negative | n.d. | n.d. | n.d. | n.d. | n.d. | 0 |  |
| **Case 24** | 0.2 | 0.57 | 0.07 | 0 | 0 | 0 | negative | 0.05 | 0 | 0 | 0 | negative | 0 |  |
| **Case 25** | 0.61 | 0.61 | 0.13 | 0.56 | 0.15 | 0.21 | negative | 0.19 | 0.02 | 0 | 0.1 | negative | 0 |  |
| **Case 26** | 0.76 | 0.66 | 0.85 | 0.98 | 0.32 | 0.68 | negative | 0 | 0.09 | 0.03 | 0.03 | negative | 80 |  |
| **Case 27** | 0.85 | 0.69 | 0.44 | 0.27 | 0.06 | 0.25 | negative | 0 | 0 | 0 | 0 | negative | 0 |  |
| **Case 28** | 0.82 | 1.52 | 1.92 | 0.63 | 0.22 | 0.52 | positive | 0.1 | 0.46 | 0.13 | 0.1 | negative | 20 |  |
| **Case 29** | 0.77 | 15.66 | 0.5 | 0.2 | 0.04 | 0.15 | negative | 0.03 | 5.68 | 1.29 | 2.06 | positive | 160 |  |

S = Spike, NCAP = Nucleocapsid protein; RBD = Receptor Binding Domain, S1 = Spike 1 domain of SARS-CoV-2, PRNT50 = plaque reduction neutralization test for neutralizing IgG antibodies; n.d. = not defined, seronegative post COVID-19 patients are highlighted in grey, seronegative post COVID-19 patients used for the T cell responses are bolded and highlighted in grey
