# Supplementary material for: Reactive T Cells in Convalescent COVID-19 Patients With Negative SARS-CoV-2 Antibody Serology
Source: Front Immunol. 2021 Jul 12;12:687449. doi: 10.3389/fimmu.2021.687449 (PMC8312095; doi:10.3389/fimmu.2021.687449)
Supplement: Supplementary file 3 [file Table_2.docx]

**Supplementary Table 2:** Detailed serological data of all seropositive convalescent individuals and healthy controls.

|  | **EUROIMMUN** | | **IgG** | | | | | **IgA** | | | | |  |
| --- | --- | --- | --- | --- | --- | --- | --- | --- | --- | --- | --- | --- | --- |
|  | **S-IgG** | **S-IgA** | **NCAP** | **RBD** | **S1** | **full spike** | **result** | **NCAP** | **RBD** | **S1** | **full spike** | **result** | **PRNT50** |
| **Ab+ 1** | 6.94 | 8.49 | 3.5 | 4.38 | 3.52 | 3.48 | positive | 0.72 | 2.53 | 0.58 | 1.36 | positive | 320 |
| **Ab+ 2** | 4.48 | 2.04 | 2.13 | 2.83 | 2 | 1.89 | positive | 0.05 | 0.26 | 0.1 | 0.08 | negative | 80 |
| **Ab+ 3** | 1.85 | 1.98 | 0.56 | 1.12 | 0.58 | 0.51 | positive | 0.13 | 0.15 | 0.08 | 0.08 | negative | 20 |
| **Ab+ 4** | 3.02 | 1.1 | 3.02 | 2.82 | 1.45 | 1.7 | positive | 0 | 0.18 | 0.05 | 0.03 | negative | 80 |
| **Ab+ 5** | 3.23 | 1.65 | 1.8 | 3.24 | 1.78 | 1.73 | positive | 0.07 | 0.24 | 0.1 | 0.15 | negative | 80 |
| **Ab+ 6** | 7.68 | 0.97 | 3.6 | 4.42 | 3.02 | 3.53 | positive | 0.08 | 0.03 | 0 | 0.13 | negative | 160 |
| **Ab+ 7** | 5.71 | 2.94 | 1.89 | 4.68 | 3.98 | 4 | positive | n.d. | n.d. | n.d. | n.d. | n.d. | 640 |
|  | | | | | | | | | | | | | |
| **HC 1** | 0.24 | n.d. | n.d. | n.d. | n.d. | n.d. | n.d. | n.d. | n.d. | n.d. | n.d. | n.d. | n.d. |
| **HC 2** | 0.24 | n.d. | n.d. | n.d. | n.d. | n.d. | n.d. | n.d. | n.d. | n.d. | n.d. | n.d. | n.d. |
| **HC 3** | 0.26 | n.d. | n.d. | n.d. | n.d. | n.d. | n.d. | n.d. | n.d. | n.d. | n.d. | n.d. | n.d. |
| **HC 4** | 0.20 | n.d. | n.d. | n.d. | n.d. | n.d. | n.d. | n.d. | n.d. | n.d. | n.d. | n.d. | n.d. |
| **HC 5** | 0.18 | n.d. | n.d. | n.d. | n.d. | n.d. | n.d. | n.d. | n.d. | n.d. | n.d. | n.d. | n.d. |
| **HC 6** | 0.17 | n.d. | n.d. | n.d. | n.d. | n.d. | n.d. | n.d. | n.d. | n.d. | n.d. | n.d. | n.d. |
| **HC 7** | 0.18 | n.d. | n.d. | n.d. | n.d. | n.d. | n.d. | n.d. | n.d. | n.d. | n.d. | n.d. | n.d. |
| **HC 8** | 0.19 | n.d. | n.d. | n.d. | n.d. | n.d. | n.d. | n.d. | n.d. | n.d. | n.d. | n.d. | n.d. |

S = Spike, NCAP = Nucleocapsid protein; RBD = Receptor Binding Domain, S1 = Spike 1 domain of SARS-CoV-2, PRNT50 = plaque reduction neutralization test for neutralizing IgG antibodies; n.d. = not defined
